# The Need for Emergency Laparotomy With Open Abdomen Therapy in the Course of ECMO—A Retrospective Analysis of Course and Outcome

**DOI:** 10.3389/fsurg.2020.00063

**Published:** 2020-09-04

**Authors:** Sissy-A. Schulz, Simone Schaefer, Dana C. Richards, Christian Karagiannidis, Panagiotis Thomaidis, Markus M. Heiss, Dirk R. Bulian

**Affiliations:** ^1^Department of Abdominal, Tumor, Transplant and Vascular Surgery, Cologne-Merheim Medical Center (CMMC), Witten/Herdecke University, Cologne, Germany; ^2^Department of Pneumology and Critical Care Medicine, ARDS and ECMO Centre, Cologne-Merheim Medical Center (CMMC), University of Witten/Herdecke, Cologne, Germany

**Keywords:** open abdomen, laparostoma, ECMO, abdominal compartment syndrome, NPWT

## Abstract

**Background:** Abdominal compartment syndrome (ACS) can occur in patients placed on extra corporeal membrane oxygenation (ECMO). This implies the necessity of decompressive laparotomy followed by an open abdomen (OA) to prevent complications such as multi-organ-failure or death.

**Methods:** We searched for ECMO patients in our hospital database between July 2015 and April 2020 and selected those with an emergency laparotomy and OA therapy. Of these, we analyzed only patients who were treated with an OA after establishing the ECMO regarding patient-related parameters like sex, age, height, weight, and indications for ECMO as well as outcome parameters like complete fascial closure rate, mortality, length of stay in intensive care unit (ICU), length and kind of OA therapy, number of surgical procedures, dressing changes concerning negative pressure wound therapy (NPWT), and number of surgical revisions.

**Results:** In eight out of 421 patients (1.9%), a laparostoma had to be created during ECMO support. For temporary closure, either NPWT, abdominal packing, or both were used. The median length of OA therapy was 17 days, and the median length of stay in ICU was 42 days in total. The median number of surgical procedures and NPWT dressing changes was seven. In three of the eight patients, a surgical revision was necessary. The total mortality rate was 50%. In 75%, the fascia could be closed. Two patients died before final closure. In all deceased patients, an abdominal packing was necessary during the course of treatment; in the survivors, only once. No enteroatmospheric fistula or abscesses occurred.

**Conclusions:** ACS in patients placed on ECMO is a very rare condition with a considerable mortality rate but high secondary closure rate of the fascia. A necessary abdominal packing due to a severe bleeding seems to be a risk factor with a potentially fatal outcome.

## Introduction

A laparostoma is a non-closure of the fascia in cases of laparotomy, which is commonly an emergency procedure. Concerning this, there are a myriad of reasons for a laparostoma, and consequently, in many cases, a tension-free closure is impossible. Laparostoma is used to restore an adequate hemodynamic status, preventing an abdominal compartment syndrome (ACS) and deferring definitive intervention and anastomosis, until the patient is hemodynamically stable and appropriately resuscitated. Early identification and draining of a residual infection are of particular importance regarding the removal of infected or cytokine-loaded fluid, and thereby the control of any persistent source of infection is facilitated by a laparostoma (1–3). Despite all of those positive aspects improving many patients' outcomes, it is also important to face the risks and complications associated with an open abdomen (OA). While some patients require further surgical procedures during their inpatient stay, others are mainly affected by long-term complications such as a remaining fascial defect, which may require further treatment (1, 4, 5). Enteroatmospheric fistulas are an example of a long-term complication in patients during or after laparostoma, as are abscesses and the loss of abdominal wall domain. These can result in an increase in morbidity and mortality (1, 5, 6).

One possible reason for an emergency laparotomy without immediate primary closure is the development of ACS. Several risk factors for developing intraabdominal hypertension (IAH) as well as ACS, like large-volume fluid resuscitation and the presence of shock, hypotension, sepsis, massive intestinal swelling, or severe trauma, are described in literature (1, 2, 6–9). In addition, patients who have had ECMO created can develop ACS without having previously suffered trauma or abdominal sepsis following abdominal surgery (10, 11).

Due to the rarity of such cases, there is very little literature describing the course and the outcome of patients who develop an ACS after the establishment of an ECMO and require an OA. Our aim was to analyze the outcome, number of days with the OA, number of days in intensive care unit (ICU), number of surgical procedures, dressing changes concerning the negative pressure wound therapy (NPWT), and number of surgical revisions in such patients admitted to our ARDS and ECMO center in Cologne-Merheim Medical Center (CMMC) comparing our results with data about laparostoma patients on ECMO described in the literature.

## Materials and Methods

### Patients

We performed a retrospective, single-center, observational cohort study of patients at the CMMC, Witten/Herdecke University teaching hospital, treated with laparostoma after the beginning of ECMO support from July 2015 to April 2020. Data were gathered from electronic medical records by searching our hospital's patient database for the ICD codes for “ECMO” and “laparostoma” / “laparotomy.” The methods for inclusion of patients and patient-related data were specified a priori.

The patients included were placed on ECMO as well as treated with laparostoma. We only included patients with laparotomy leading to an OA after the initiation of ECMO to analyze a more homogeneous group.

The patient files were screened using the parameters mentioned below.

### Definition of IAH and ACS

Intraabdominal pressure is defined as the steady-state pressure concealed within the abdominal cavity. In critically ill adults, it is ~5–7 mmHg. IAH is defined as an intraabdominal pressure of more than 12 mmHg and is classified in four grades (grade I: 12–15 mmHg, grade II: 16–20 mmHg, grade III: 21–25 mmHg, and grade IV: >25 mmHg) (3). In contrast to that, the ACS presupposes per definition a new organ dysfunction and hypertension with a pressure of more than 20 mmHg within the abdominal cavity (3, 12).

### Surgical Standard

In our hospital, laparotomy is performed at the point of an intraabdominal pressure of 20 mmHg or above, combined with clinical symptoms of ACS as anuria or insufficiency of perfusion through ECMO.

The standard procedure monitoring IAP in our patients at risk in the ICU was the measurement of pressure within the bladder 30–60 s after the instillation of 25 ml of normal saline through the urinary catheter every 8 h.

Methods of creating an OA at our hospital:

1. Applying NPWT with PU foam and visceral protective film underneath with or without redressing fasciorrhaphy.
2. Interposition of a Vicryl-mesh onto the visceral protective film for the redression of the fascia instead of redressing sutures and usage of a commercially NPWT set.

Other kinds of therapy like the Wittman patch or the Bogota bag were not deployed in our hospital. The choice of wound closure depended on the surgeons' preference.

The standard suction magnitude was 75 or 80 mmHg. Dressing changes for NPWT were performed every 3 days at our hospital.

Depending on the hemodynamical stability of the patient, the surgical creation of the laparostoma was either performed in our central operation room or in ICU.

### Outcome Parameter

The outcome parameters of the study were patient-related parameters like sex, age, height, weight, and indications for ECMO as well as outcome parameters like successful fascial closure rate, mortality, length of stay in ICU after closure and in total, length and kind of OA therapy, number of surgical procedures performed, number of dressing changes concerning NPWT, and number of surgical revisions.

### Statistics

The data were prepared and analyzed in Microsoft Excel Version 14.1.0 (Microsoft Corp., Redmond, WA, United States). Data of continuous variables are expressed as minimum, maximum, and median. Binary and categorical variables are reported as counts and percentages.

## Results

Between July 2015 and April 2020, we treated 421 patients on ECMO in total. Among these, we identified 14 patients who underwent decompressive laparotomy followed by the state of an OA (8/421; 1.9%). In our analysis, only patients on ECMO who developed ACS after cannulation were included (8/14; 57%). Table 1 depicts data of these patients. All eight patients were supported by veno-venous ECMO; one of them (12.5%) initially was placed on veno-arterial ECMO, which was converted within the 1st day of the ECMO support.

**Table 1:** Patient related data.

**Patient**	Sex^*^	Age^**^	**Height (cm)**	**Weight (kg)**	**BMI (kg/m^**2**^)**	**Indications for ECMO**
1	-	11–15	162	60	22.9	ARDS after pulmonary aspiration during CPR
2	-	31–35	185	85	24.0	Secondary ARDS (severe pancreatitis)
3	-	61–65	160	70	26.7	COPD
4	-	66–70	180	80	24.7	Thoracic trauma
5	-	61–65	178	92	29.0	Secondary ARDS due to severe pancreatitis
6	-	76–80	165	95	34.9	Ruptured pars membranacea (trachea) while tracheotomy
7	-	51–55	160	65	25.4	Viral pneumonia, (Influenza A, H1N1)
8	-	36–40	163	89	33.5	Bilateral, nosocomial, bacterial pneumonia
In total	*m* = 4 (50%) *f* = 4 (50%)^***^	56 (14–77)^****^	164 (160–180)^****^	82.5 (60–95)^****^	26.1 (22.9–34.9)^****^	

* *Only summarized data*.

** *Presenting as a range (11–15, 16–20, 21–25, 26–30.)*.

*** *Counts (Percentage)*.

**** *Median (Min–Max)*.

*m, Male; f, Female; BMI, Body mass index; ECMO, Extra corporeal membrane oxygenation; ARDS, Acute respiratory distress syndrome; CPR, Cardio pulmonary resuscitation; COPD, Chronic obstructive pulmonary disease*.

The median age of our male patients was lower than that in our female patients (46.5 vs. 56.0 years).

Outcome data are depicted in Table 2. Two of the deceased patients died with a non-closed abdomen (days 5 and 7 of the OA). The other two patients died 1 and 16 days after abdominal closure.

**Table 2:** Outcome parameters.

**Patient no**.	**Deceased**	**Days on ICU**	**Days with OA**	**Days on ICU after closure**	**Number of surgical procedures**	**Abdominal packing**	**Negative pressure wound therapy**	**Number of dressing changes for NPWT**	**Number of revisions**
1	No	66	33	33	15	Yes	Yes	11	1
2	No	73	71	1	26	No	Yes	23	1
3	Yes^*^	115	9	16	4	Yes	Yes	2	0
4	Yes^*^	12	12	12	5	Yes	Yes	4	0
5	No	41	28	23	10	No	Yes	8	0
6	No	43	22	17	9	No	Yes	7	0
7	Yes^**^	25	5	n.a.	2	Yes	Yes	0	0
8	Yes^**^	30	7	n.a.	2	Yes	No	n.a.	1
In total	•No = 4 (50%) •Yes = 4 (50%)^***^	42 (12–115)^****^	17 (5–71)^****^	16.5 (1–33)^****^	7 (2–26)^****^	•No = 3 (37.5%) •Yes = 5 (62.5%)^***^	•No = 1 (12.5%) •Yes = 7 (87.5%)^***^	7 (0–23)^****^	0 (0–1)^****^

* *Death after fascial closure*.

** *Death before fascial closure*.

*** *Counts (Percentage)*.

**** *Median (Min–Max)*.

*ICU, Intensive care unit; OA, Open abdomen; NPWT, Negative pressure wound therapy*.

The median age of surviving patients was lower than the median age of the patients who did not (46.5 vs. 56 years); 87.5% (7/8) of the ECMO patients with an OA were treated with NPWT. In 62.5% (5/8), abdominal packing was implemented initially or during the course. One patient was only treated with abdominal packing. In all eight patients, we did not use any other techniques of temporary closure than NPWT or abdominal packing. Observing the four surviving patients, the median duration of laparostoma was 30.5 days. The median number of abdominal surgeries in the patients who survived until final closure (6/8; 75%) was 12.5; the median number of dressing changes during NPWT was 9.5. In three patients, relaparotomy was required. One was due to ACS a few hours after the first attempt of closure. The second was a planned exploration after initial emergency surgery, and the third was due to acute bleeding. In all four patients who did not survive, abdominal packing had to be applied (100%), whereas only one of the four surviving patients (25%) was treated with abdominal packing. In two patients (25%), retroperitoneal hemorrhage appeared, while in one patient, it was after initial trauma and thus not caused by the placement on ECMO; in the second, it occurred after the initiation of ECMO support. All abdominal findings during the initial laparotomy were operable.

The median length of stay in ICU of the surviving patients was 42 days, and the median length of stay in ICU after closure was 20 days.

In six patients (6/8; 75%), laparotomy was the initial procedure to relieve the elevated intraabdominal pressure, once ACS has been diagnosed. In two patients (2/8; 25%), draining of fluids was performed previously by puncture.

In all patients whose OA was finally closed, secondary wound closure was performed in layers. We did not perform closure with a partial defect of the abdominal wall or the usage of a mesh. Complications during the OA such as enterocutaneous fistulas or abscesses could not be found.

## Discussion

The huge impact of an increased intraabdominal pressure can be seen in the impaired functions of multiple organs such as the lungs, bowel, and the kidneys. That is why immediate diagnosis and appropriate intervention is of vital concern (8, 9, 12–14). This ranges from medical treatment of IAH to surgical treatment when the patient's condition aggravates or ACS develops (14). To relieve the excess pressure, decompressive laparotomy, which represents a non-anatomical situation, is considerable and leads to an OA in many cases. Other causes for an OA are trauma, the effects of abdominal sepsis, also leading to increased abdominal pressure, and damage control surgery (1, 6, 15, 16). During this surgical treatment, with an increased morbidity and mortality, the loss of fluids and temperature, as well as the desiccation of the bowels, must be considered (6).

Multiple ways to manage an OA using temporary or, if possible, final closing techniques are described in the literature. Most of the temporary abdominal closure techniques, providing protection to the abdominal viscera during the time the fascia remains open, include negative pressure therapy techniques such as vacuum packing and vacuum-assisted therapy (1, 17–19). One major objective over the course of laparostoma is to prevent the fascia from retraction, which leads to the impossibility of final closure of the fascia. To prevent this or even to redress the fascia gradually, a mesh can be sewn into the defect. Other options are the sandwich and zipper technique, as well as the artificial burr device or the Wittmann Patch. Although these techniques finally provide a high primary closure rate, they may lead to a remaining gap in the fascia (17, 20–22). Some authors describe the Bogota bag or dynamic retention sutures to be considerable options for temporary closure of the OA (17, 23). In general, closure is recommended to be achieved at the earliest expected time (1).

Depending on the cause for an OA in a given case, distinct conditions can impair primary abdominal closure. These include visceral edema, the inability to control a source of infection, the necessity for second-look surgery or completion of previous treatment, and severe cases of abdominal wall damage, especially given in patients with penetrating trauma (15, 24). Patients who fail primary closure may require a biologic fascial bridge with subsequent fascia repair in the future (4).

In patients on ECMO, IAH can be detected by a reduced flow in the return canula and generates end-organ malperfusion. That is why ACS, followed by the performance of decompressive laparotomy, should be considered in cases of hemodynamic impairment or ECMO dysfunction, to diminish complications (10, 11).

There is a paucity of literature about the development of an ACS followed by laparostoma in ECMO patients, even though ECMO is a risk factor for an increased intraabdominal pressure. Due to the low number of cases, information about this subject, especially about the duration of an OA including the number and kind of surgical procedures in patients on ECMO, is rare. This study gives an insight into the courses of disease for our patients and their outcomes. We found a mortality rate of 50% and a final closure rate of 75% (25% died before, 25% after final closure). Only one patient (12.5%) required an unpredictable surgical procedure on account of a major bleeding event. The techniques used for temporary closure were NPWT and abdominal packing. According to our research, abdominal packing seems to be a risk factor with a potentially fatal outcome. As the OA in ECMO patients is known to be a rare condition, the number of patients we found was correspondingly not high enough to gather universally valid information, but to provide data of a barely investigated thematic area.

In the literature, we found a few studies referring to related substances. McCann et al. performed a retrospective, single-center cohort study with 355 patients on ECMO in 2019 (25). The prevalence of emergency laparotomy in this study was 3.7% (13/355). In six patients (6/13), the abdomen was closed in the same procedure; in two patients (2/13), NPWT was used; and in five patients (5/13), the Bogota bag was used. The mortality rate was 69% among patients with emergency laparotomy until hospital discharge. They described intraoperative major hemorrhage to be rare (2/13; 15.4%). Among our patients, two cases of bleeding could be identified, of which none was intraoperative. One was due to initial trauma and one was during ECMO support. In 2018, Glowka et al. analyzed 175 patients who underwent ECMO support. Eleven out of 175 patients developed an ACS and underwent decompressive laparotomy (11/175; 6.3%). In four of these patients (4/11), ECMO support was performed as veno-venous, and in seven patients (7/11), it was veno-arterial. Eight of them (8/11; 72%) died while in the hospital, and age was described as a risk factor (15). In comparison, the prevalence of laparotomy and the mortality in our patient group was lower (1.9 vs. 3.7% and 6.3%; 50 vs. 69% and 72%). As our median patient age was lower in the ones who survived, we also suggest age to be a risk factor for a fatal outcome. In all of our patients who did not survive, abdominal packing was performed. That is why we believe abdominal packing to be a risk factor with a potentially fatal outcome. However, there is also a bias, since patients requiring abdominal packing are usually in a worse condition than those who do not. The low number of eligible patients in all these studies including our analysis is an indication of the rare incidence of ACS in this patient group rather than a significant quality of medical therapy.

We did not find any literature giving a more precise indication about the state of an OA from the surgical point of view, like type, duration, and number of surgical procedures in patients on ECMO. Since none of our patients has had any kind of outpatient follow-up after the inpatient stay, our study cannot describe any long-term complications after the abdominal closure.

## Conclusion

The development of ACS, leading to the necessity of decompressive laparotomy followed by an OA, is a rare complication of patients on ECMO support, but has a relevant mortality. On the other hand, the secondary closure rate of the fascia is very high. The need of abdominal packing seems to be a risk factor for a fatal outcome. However, the small number of our includable eight patients limits any conclusion. Accordingly, a prospective multicenter study with more patients is necessary to confirm our results.

## Data Availability Statement

All datasets generated for this study are included in the article/supplementary material.

## Author Contributions

S-AS, SS, and DB conceived the idea and designed this study. S-AS, SS, PT, and DB did the statistical analysis and drafted the manuscript. DR, CK, and MH contributed to the data interpretation and critically revised the manuscript. All authors have seen and approved the final manuscript.

## Conflict of Interest

The authors declare that the research was conducted in the absence of any commercial or financial relationships that could be construed as a potential conflict of interest.

## References

[1] Sartelli M Abu-Zidan FM Ansaloni L Bala M Beltran MA Biffl WL. The role of the open abdomen procedure in managing severe abdominal sepsis: WSES position paper. World J Emerg Surg. (2015) 10:35. 10.1186/s13017-015-0032-7 26269709 PMC4534034

[2] Rollins MD Deamorim-Filho J Scaife ER Hubbard A Barnhart DC. Decompressive laparotomy for abdominal compartment syndrome in children on ECMO: effect on support and survival. J Pediatr Surg. (2013) 48:1509–13. 10.1016/j.jpedsurg.2012.10.052 23895964

[3] Kirkpatrick AW Roberts DJ De Waele J Jaeschke R Malbrain ML De Keulenaer B. Intra-abdominal hypertension and the abdominal compartment syndrome: updated consensus definitions and clinical practice guidelines from the World Society of the Abdominal Compartment Syndrome. Intensive Care Med. (2013) 39:1190–206. 10.1007/s00134-013-2906-z 23673399 PMC3680657

[4] Regner JL Kobayashi L Coimbra R. Surgical strategies for management of the open abdomen. World J Surg. (2012) 36:497–510. 10.1007/s00268-011-1203-7 21847684

[5] Balogh ZJ Leppaniemi A. Patient populations at risk for intra-abdominal hypertension and abdominal compartment syndrome. Am Surg. (2011) 77(Suppl.1):S12–6. 21944446

[6] Shanmugakrishnan RR Loh CYY Wakure A El-Muttardi N. Serial abdominal closure with Gore-tex mesh and Rives-Stoppa for an open abdomen secondary to intra-abdominal hypertension in burns. Indian J Plast Surg. (2018) 51:324–6. 10.4103/ijps.IJPS_75_18 30983735 PMC6440362

[7] Holodinsky JK Roberts DJ Ball CG Blaser AR Starkopf J Zygun DA. Risk factors for intra-abdominal hypertension and abdominal compartment syndrome among adult intensive care unit patients: a systematic review and meta-analysis. Crit Care. (2013) 17:R249. 10.1186/cc13075 24144138 PMC4057241

[8] Coccolini F Roberts D Ansaloni L Ivatury R Gamberini E Kluger Y. The open abdomen in trauma and non-trauma patients: WSES guidelines. World J Emerg Surg. (2018) 13:7. 10.1186/s13017-018-0167-4 29434652 PMC5797335

[9] Rogers WK Garcia L. Intraabdominal hypertension, abdominal compartment syndrome, and the open abdomen. Chest. (2018) 153:238–50. 10.1016/j.chest.2017.07.023 28780148

[10] Augustin P Lasocki S Dufour G Rode J Karsenti A Al-Attar N. Abdominal compartment syndrome due to extracorporeal membrane oxygenation in adults. Ann Thorac Surg. (2010) 90:e40–1. 10.1016/j.athoracsur.2010.06.039 20732475

[11] Boulos FM Pasrija C DiChiacchio L Rouse M Raithel M Mackowick K. Early decompressive laparotomy for intra-abdominal hypertension following initiation of venovenous extracorporeal membrane oxygenation. Asaio J. (2019) 66:520–3. 10.1097/MAT.0000000000001045 31425255

[12] Bailey J Shapiro MJ. Abdominal compartment syndrome. Crit Care. (2000) 4:23–9. 10.1186/cc646 11094493 PMC137249

[13] Sun K Hancock BJ Logsetty S. Ischemic bowel as a late sequela of abdominal compartment syndrome secondary to severe burn injury. Plast Surg. (2015) 23:218–20. 10.1177/229255031502300407 26665133 PMC4664133

[14] Nirula R. Two case studies of cardiopulmonary effects of intra-abdominal hypertension. Surg Clin North Am. (2012) 92:1679–84. 10.1016/j.suc.2012.08.017 23153890

[15] Glowka TR Schewe JC Muenster S Putensen C Kalff JC Pantelis D. Decompressive laparotomy for the treatment of the abdominal compartment syndrome during extracorporeal membrane oxygenation support. J Crit Care. (2018) 47:274–9. 10.1016/j.jcrc.2018.07.024 30096634

[16] Godat L Kobayashi L Costantini T Coimbra R. Abdominal damage control surgery and reconstruction: world society of emergency surgery position paper. World J Emerg Surg. (2013) 8:53. 10.1186/1749-7922-8-53 24341602 PMC3878509

[17] Kreis BE de Mol van Otterloo AJ Kreis RW. Open abdomen management: a review of its history and a proposed management algorithm. Med Sci Monit. (2013) 19:524–33. 10.12659/MSM.883966 23823991 PMC3706408

[18] Leppäniemi AK. Laparostomy: why and when? Critical Care. (2010) 14:216. 10.1186/cc8857 20236460 PMC2887109

[19] Davis J Caruso DM Foster KN Matthews MR. A novel approach to sealing the denuded dermis of the abdominal wall with a negative pressure wound device after a decompressive laparotomy. J Burn Care Res. (2018) 39:838–42. 10.1097/BCR.0000000000000615 28661985

[20] Schein M Saadia R Jamieson JR Decker GA. The 'sandwich technique' in the management of the open abdomen. Br J Surg. (1986) 73:369–70. 10.1002/bjs.1800730514 3708284

[21] Keramati M Srivastava A Sakabu S Rumbolo P Smock M Pollack J. The Wittmann Patch s a temporary abdominal closure device after decompressive celiotomy for abdominal compartment syndrome following burn. Burns. (2008) 34:493–7. 10.1016/j.burns.2007.06.024 17949916

[22] Koss W Ho HC Yu M Edwards K Ghows M Tan A. Preventing loss of domain: a management strategy for closure of the “open abdomen” during the initial hospitalization. J Surg Educ. (2009) 66:89–95. 10.1016/j.jsurg.2008.12.003 19486872

[23] Muhammad Y Gondal KM Khan UA. Use of the “bogota bag” for closure of open abdominal wound after exploratory laparotomy—our experience at Mayo Hospital Lahore. J Pak Med Assoc. (2016) 66:980–3. 27524532

[24] Beale EW Janis JE Minei JP Elliott AC Phelan HA. Predictors of failed primary abdominal closure in the trauma patient with an open abdomen. South Med J. (2013) 106:327–31. 10.1097/SMJ.0b013e31829243ed 23644642

[25] McCann C Adams K Schizas A George M Barrett NA Wyncoll DLA. Outcomes of emergency laparotomy in patients on extracorporeal membrane oxygenation for severe respiratory failure: a retrospective, observational cohort study. J Crit Care. (2019) 53:253–7. 10.1016/j.jcrc.2019.06.021 31301640

